# Crypto-Currency: Investing in New Models to Advance the Study of *Cryptosporidium* Infection and Immunity

**DOI:** 10.3389/fcimb.2020.587296

**Published:** 2020-11-18

**Authors:** N. Bishara Marzook, Adam Sateriale

**Affiliations:** Cryptosporidiosis Laboratory, The Francis Crick Institute, London, United Kingdom

**Keywords:** Parasites (Apicomplexa), host-pathogen, intestinaI epithelial barrier, *Cryptosporidium*, intestinal immunity, organoid culture, mouse model

## Abstract

Cryptosporidiosis is a leading cause of diarrheal disease and an important contributor to global morbidity and mortality. Although the brunt of disease burden is felt by children in developing countries, *Cryptosporidium* is a ubiquitous intestinal parasite with frequent outbreaks around the world. There are no consistently effective treatments for cryptosporidiosis and the research to drive new developments has stagnated, largely due to a lack of efficient *in vivo* and *in vitro* models. Fortunately, these research barriers have started to fall. In this review, we highlight two recent advances aiding this process: A tractable mouse model for *Cryptosporidium* infection and stem cell-based *in vitro* culture systems that mimic the complexity of the host intestine. These models are paving the way for researchers to investigate *Cryptosporidium* infection and host immunity down to a molecular level. We believe that wise investments made to adopt and develop these new models will reap benefits not only for the *Cryptosporidium* community but also for the intestinal immunology field at large.

## Introduction

**Figure 1:**
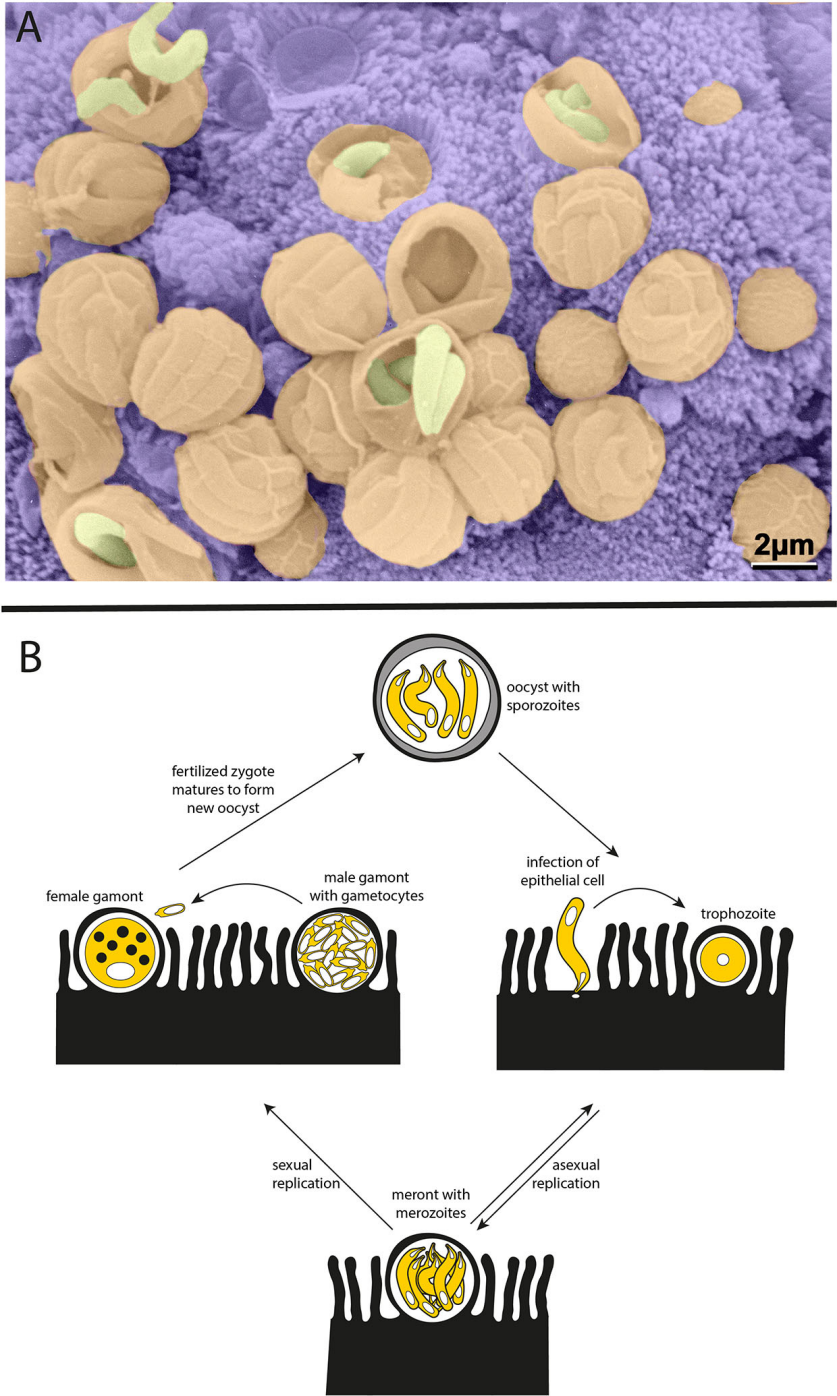
*Cryptosporidium* parasites. **(A)** Parasitophorous vacuoles line the wall of the small intestine in an infected mouse. Scanning electron microscopy with parasites in yellow, parasitophorous vacuoles in orange, and host intestinal villi in purple (image courtesy of Prof. David Ferguson, Oxford University, copyright retained). **(B)** Life cycle of the *Cryptosporidium* parasite. Each oocyst contains four sporozoites that can infect the epithelium lining the host intestinal tract. Sporozoites mature to trophozoites and then meronts. Meronts release merozoites which go on to reinvade nearby epithelial cells. The switch from asexual to sexual replication during the *Cryptosporidium* life cycle is poorly understood and remains an area of great interest. During sexual replication, male and female gametes are produced, and fertilization leads to the formation of new infectious oocysts.

*Cryptosporidium* is a member of the apicomplexan phylum of parasites and a distant relative of *Plasmodium*, the causative agent of malaria. Unlike *Plasmodium*, *Cryptosporidium* is monoxenous—completing its entire life cycle within a single host—and infection is specifically localized to the gastrointestinal tract. The infectious form of the parasite, known as the oocyst, is resistant to a variety of standard disinfectants, including chlorination. Each *Cryptosporidium* oocyst harbors four sporozoites, which are released when oocysts are ingested by a host and invade the apical side of polarized intestinal epithelial cells. Parasitophorous vacuoles can be readily seen lining the intestine of an infected host (**Figure 1A**). *Cryptosporidium* then undergo asexual and sexual cycles of replication, leading to the production of new oocysts that are shed by the infected host (**Figure 1B**).

Human cryptosporidiosis is mainly caused by two species: the anthroponotic *Cryptosporidium hominis* and the zoonotic *Cryptosporidium parvum*. The pathology of a *Cryptosporidium* infection manifests as diarrhoeal disease, particularly in children in low-income countries during the first 2 years of life (Kotloff et al., 2013). While severe cases can be fatal, the majority of *Cryptosporidium* infections resolve within a few weeks. Because of this self-limiting nature, it is tempting to focus solely on acute symptoms and short-term consequences. However, infection in young children is closely associated with bouts of prolonged malnutrition that lead to important long-term consequences, such as impaired growth and cognitive development (Korpe et al., 2016). A recent re-evaluation of the *Cryptosporidium* disease burden estimates an annual global loss of nearly 13 million disability adjusted life years; a figure largely driven by malnutrition associated morbidity (Khalil et al., 2018). Despite this substantial disease burden, there are no consistently effective treatments for cryptosporidiosis. Nitazoxanide, the only FDA-approved drug to treat infection, has limited efficacy in the immunocompetent and performs on par with placebo in immunocompromised or malnourished patients (Amadi et al., 2002). Development of new therapeutics has been severely hindered by a lack of effective *in vitro* and *in vivo* models for *Cryptosporidium* infection. However, recent advances in these fields are very promising, with novel platforms for *Cryptosporidium* research that allow for infection and immunity to be studied in more physiologically-relevant settings.

## *In Vivo*: A Genetically Tractable Natural Mouse Model

**Figure 2:**
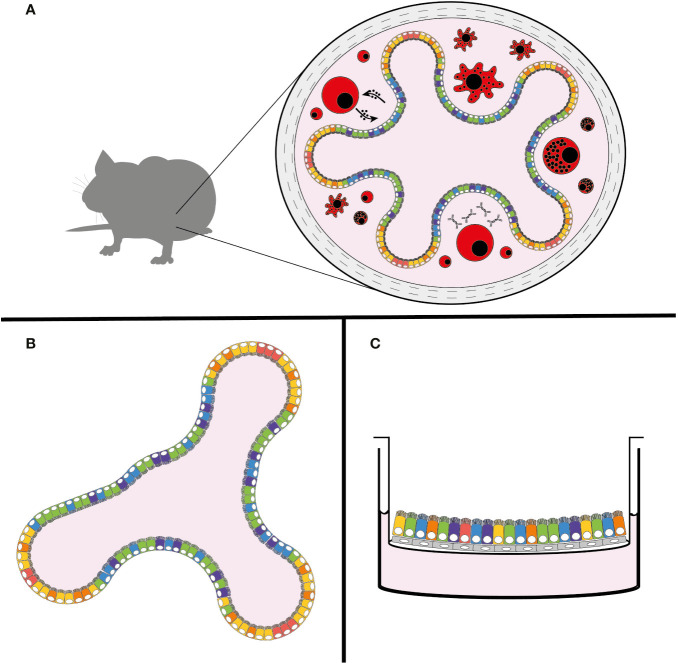
New models of *Cryptosporidium* infection. **(A)** Natural mouse model of cryptosporidiosis, **(B)** intestinal organoid model, and **(C)** air-liquid interface (ALI) model. The use of colours is meant to demonstrate the complexity and diversity of cell types that are present in these models, with the natural mouse model including the host immune response (in red).

The genus *Cryptosporidium* infects a broad range of vertebrate hosts from humans to fish. Despite this wide range of hosts, animal models that recapitulate human infection are historically lacking. Calves, gnotobiotic piglets, and immunocompromised mice have all been used to model infection with varying degrees of success. These *in vivo* models are still the gold standard of *Cryptosporidium* infection studies, since they provide the unique dynamic environment of the host intestine, its complex tissue architecture, diversity of cell types, and host immune responses (**Figure 2A**). However, due to the inability to adequately further manipulate the genetics of these host organisms, and the high costs associated with their upkeep, researchers have been unable to explore mechanisms of the immune response to *Cryptosporidium*. In an effort to overcome these restrictions, *Cryptosporidium tyzzeri*, a species of the parasite that naturally infects mice, has recently been developed as a genetically tractable model of human infection (Sateriale et al., 2019).

*C. tyzzeri* derives its name from EE Tyzzer, the researcher who first discovered *Cryptosporidium* as a natural parasite infection of laboratory mice at the turn of the 20^th^ century. Tyzzer named the parasites that he found in the mouse small intestine *Cryptosporidium parvum*, a Latin reference to its morphology (cryptosporidium, ‘hidden spore’) and size (parvum, ‘small’). Over the next century, ‘*Cryptosporidium parvum*’ was continually rediscovered in the intestines of many species, including cows (Slapeta, 2011). With the help of improved genetic analyses, it was later determined that these new isolates of ‘*Cryptosporidium parvum*’ are, in fact, many separate species of the parasite. Despite efforts to rescue the original nomenclature, the taxonomic name *Cryptosporidium parvum* currently refers to the parasite species that is a natural infection of cows, and *Cryptosporidium tyzzeri* refers to the natural mouse pathogen that Tyzzer likely discovered (Ren et al., 2012). Now recognized as a ubiquitous infection of the common house mouse, *Mus musculus*, the *C. tyzzeri* parasite appears to be co-evolving with the various subspecies of its murine host (Kváč et al., 2013).

*C. tyzzeri* is a very close relative of the human-infecting parasite species; genetically 95-96% identical at the nucleotide level to *C. hominis* and *C. parvum*. Most importantly, *C. tyzzeri* infection in mice mimics human disease in pathology and immune response. Cryptosporidiosis is a common infection among patients with HIV/AIDS, and the disease can be protracted and severe. Because there are no effective drug treatments, resolution of infection is closely tied to the recovery of normal CD4 T-cell counts (Wang et al., 2018). In the murine model, mice lacking T-cells suffer from protracted and severe infections with *C. tyzzeri*. Mice lacking B-cells, however, show no protracted illness and clear the parasite just as well as wild-type mice (Sateriale et al., 2019). In humans, patients that produce insufficient amounts of the cytokine interferon gamma (IFN*γ*) are documented to be highly susceptible to cryptosporidiosis (Morales et al., 1996). In mice, IFN*γ* deficiency also leads to a much higher parasite burden that is controlled but never cleared (Griffiths et al., 1998; Sateriale et al., 2019).

Genetic modification of the *C. tyzzeri* genome is possible through adaptation of CRISPR/Cas9-driven homologous recombination. Originally pioneered in *C. parvum* (Vinayak et al., 2015), this technique can be used to derive stable transgenic *Cryptosporidium* strains expressing luminescent and fluorescent reporters for quantitative studies of mouse infections, and mutant strains for the study of particular parasite genes. For those genes that are found to be essential, or for the temporal control of protein expression, the newly developed technique of conditional protein degradation in *Cryptosporidium* should prove very useful (Choudhary et al., 2020). With *C. tyzzeri*, this ability to genetically manipulate the parasite can be leveraged with the variety of mouse species and strains available to researchers to allow for host-pathogen interactions to be rigorously interrogated at a new level.

## *In Vitro*: Stem Cell-Based Cell Culture Models

The most widely used cell culture model for *Cryptosporidium* infection is the ileocecal adenocarcinoma cell line HCT-8 (Upton et al., 1994). This cell line has served as the primary infection model for anti-cryptosporidial drug screening and has provided many important insights into the biology of infection (Love et al., 2017; Jumani et al., 2019; Pawlowic et al., 2019). However, HCT-8s and the standard 2-D model of infection is restrictive in two crucial aspects: 1) Cell monolayers lack the cell diversity and architecture of the host intestine that is important for investigating pathogenesis, and 2) parasite growth is short-lived in this system as they are unable to complete the sexual stage of their life cycle. Recent research has implicated this sexual cycle, specifically the inability of male parasite gametes to fertilize female gametes, as the restrictive stage in the 2-D cell monolayer infection model (Tandel et al., 2019). This breakdown of fertilization is hypothesized to cause the arrested parasite growth that occurs around 72 hours after infection of HCT-8s (and other 2-D epithelial cell models). However, several *in vitro* systems have been able to sustain long-lived *Cryptosporidium* infections by recapitulating conditions that more closely mimic the host intestine.

### Intestinal Organoid Model

The developing organoid model system of *in vitro* cell culture has been a game-changer for stem cell and cancer biologists, with infectious disease studies following closely behind [for a comprehensive review see Rossi et al. (2018)]. In brief, organoids are three-dimensional growths arising from embryonic, induced pluripotent, or adult stem cells. When given proper differentiation signals and an extracellular matrix (ECM)-derived scaffold, stem cells can develop into avatars of the desired organ. Organoids from adult stem cells recapitulate most of the architecture and cell types characteristic of the tissue from which they were derived and hence can be used to study a range of aspects including basic cell and tumor biology, organ regeneration, and increasingly host-pathogen interactions in a more physiologically relevant setting. Organoids of many different systems including the brain, liver, and lung have been successfully created; however, arguably the most well-established and studied platform is that of the intestinal organoid, which has been well-characterized from both mice and men (Sato et al., 2009). In a mixture of ECM proteins, small intestinal crypt-derived stem cells self-organize into mini-organs consisting of differentiated enterocytes and secretory cells, while still maintaining intestinal stem cells, a strict apical-basal polarity, and basic crypt-villus architecture (**Figure 2B**) (Sato et al., 2009).

Recently, Heo *et al.* were able to deploy human small intestinal (SI) and lung organoid systems to model a *C. parvum* infection (Heo et al., 2018). Transmission electron microscopy (TEM) showed the presence of asexual parasite stages 1 day post-infection, as well as the presence of sexual stages and oocysts after 5 days. TEM of infected organoids was also able to indicate that enterocytes in SI organoids, but both secretory and non-secretory cells in lung organoids, were predominantly infected by *Cryptosporidium*. New oocysts produced in organoids were capable of maintaining productive infections over three rounds of serial passage in SI organoids, however the authors noted a reduction in parasite numbers over 4 weeks of passaging. High-throughput RNAseq analysis of infected organoids revealed the activation of an enterocyte-based interferon response. Analysis of parasite RNA expression also demonstrated the upregulation of oocyst wall genes (especially COWP1) at 72 hours in both SI and lung organoids, a finding that supports the observation of new oocyst generation in this system. It should be noted, however, that female gametes produce oocyst wall proteins even in the absence of fertilization from male gametes (Tandel et al., 2019). Male or microgamete specific genes such as HAP2 were not identified as being upregulated in this study. One of the features of SI organoids is the possibility of maintaining them in either ‘expanding’ (more undifferentiated stem cells and highly proliferative progenitor cells) or ‘differentiating’ (more terminally differentiated cells) states, depending on which growth factors are provided. Interestingly, *C. parvum* propagation was on average almost ten times higher in differentiated organoids compared to expanding ones.

### Air-Liquid Interface (ALI) Model

Originally devised for the culture of primary nasal epithelial cells, another recently-developed system to create differentiated intestinal cells from stem cells is the air-liquid interface (ALI) culture (**Figure 2C**) (Wang et al., 2015). Here, stem cells (either from primary sources or previously maintained as spheroids) can be plated onto 2-D membrane-supported cell culture inserts with or without an ECM-derived gel base. A layer of irradiated fibroblasts can also be used as feeder cells. Removal of liquid media from the top of the inserts results in the differentiation of stem cells into enterocyte and secretory cells. This technique was recently adapted by Wilke *et al.* to successfully infect differentiated mouse intestinal epithelial cells with *C. parvum* (Wilke et al., 2019). Parasite growth monitored by quantitative PCR showed amplification by nearly 100-fold over 20 days. Similar to the organoid model, ALI cultures were capable of producing new infectious oocysts 3 days post-infection; oocysts were capable of infecting both mice and new membrane-supported cell monolayers, albeit with diminishing returns. Also similar to the organoid model, TEM showed the preferential infection of enterocytes, however some infected secretory cells were also reported. Interestingly again, *C. parvum* grew significantly better in differentiated ALI cells.

Using CRISPR/Cas9-based genetic manipulation, Wilke *et al.* were also able to demonstrate meiotic recombination within the ALI system with fluorescently labelled parasite lines (Wilke et al., 2019). This meiotic recombination event, which naturally occurs in the final stages of the *Cryptosporidium* life cycle, appears in their ALI model after the third day of infection and coincides with the production of new infectious oocysts. The ability to perform controlled genetic crosses of *Cryptosporidium* parasites using a cell culture platform is a major advancement for the field. Exploring the nature and frequency of recombination events and how they contribute to pathogenicity and ongoing evolution of *Cryptosporidium* will greatly further the study of this deadly parasite.

## Discussion

Children living in endemic regions are particularly vulnerable to *Cryptosporidium* infection and this is well evidenced in large scale studies of diarrheal aetiology. In the Global Enteric Multicenter (GEM) study, with a cohort of over 20,000 children across Africa and southeast Asia, *Cryptosporidium* was identified as one of the primary causes of severe diarrheal events (Kotloff et al., 2013). However, from the third year onward, cryptosporidiosis becomes a negligible infection with very few attributable cases of severe disease reported in the GEM study. This apparent resistance in older children and adults in endemic regions is likely to be a matter of acquired immunity rather than age; *Cryptosporidium* is a common cause of travellers’ diarrhoea in adults traveling from non-endemic regions. Early results using *C. tyzzeri* to model human infection support these observations. Mice that recover from a natural *C. tyzzeri* infection show resistance to subsequent infections (Sateriale et al., 2019). Vaccination using irradiated parasites had the same effect of protecting against subsequent infection and disease. Unlike its relative *Plasmodium*, *Cryptosporidium* appears to lack the large gene families required for antigenic switching (Abrahamsen et al., 2004). This may be a great advantage for development of a vaccine, yet researchers first need to understand the mechanism and markers of *Cryptosporidium* immunity.

The true power of this newly developed mouse model of cryptosporidiosis lies in the ability to genetically manipulate both the host and parasite. With *C. tyzzeri*, researchers can branch out from the few immunocompromised mouse strains that are traditionally used for *Cryptosporidium* infection studies. There is a vast range of mouse species, strains, congenics and transgenics to explore pathogenesis. Next generation panels of recombinant inbred lines, such as the Collaborative Cross, can be leveraged for genome-wide interrogation of the host response to *Cryptosporidium* infections (Aylor et al., 2011). This new mouse model of infection holds the potential to study both innate and adaptive mechanisms of immunity and, coupled with stem cell-based *in vitro* culture, microscopic observations can be studied in controlled environments.

Both the organoid and ALI culture systems raise the possibility of conducting *in vitro* studies of immune cell interaction with *Cryptosporidium*-infected epithelial cells. Co-culture studies of infected epithelial cells with immune cells have already proven successful for other host-pathogen studies [for a comprehensive review see Bar-Ephraim et al. (2019)], and therefore could enhance our understanding of how the immune system detects and responds to a *Cryptosporidium* infection. There are also many areas of molecular immunology that are poorly recapitulated in the 2-D cell culture systems. Although it has been known for nearly 30 years that IFN*γ* is a crucial cytokine for resistance to *Cryptosporidium*, the particular mechanism of this cytokine’s effect on *Cryptosporidium* infection remains largely a mystery. It is likely that stem cell-based models will allow for the study of these immune mechanisms in a finely tuneable system that closely resembles the infected intestine.

Another key feature of stem cell-based parasite propagation systems is that they allow the presence of all cell types normally present in the gut (often arising from a single stem cell), and hence they provide us with the opportunity to better understand the particular intestinal niche of this obligate parasite, as well as observe any effects it might exert on existing host cell dynamics and populations over time. One of the open questions in the field is why infections of traditional 2-D cell culture systems don’t lend themselves to the generation of new oocysts. Recent work in standard HCT-8 culture has elegantly shown that this process is likely stalled at the stage of male gametocyte penetration into the female macrogamont (Tandel et al., 2019). However, this block is overcome in the ALI cultures which are still technically 2-D but involve more polarised and differentiated epithelial cells (Wilke et al., 2019). Changes in host cell metabolism, such as an upregulation of genes involved in oxidative phosphorylation, with a concomitant downregulation of the glycolytic pathway, was also associated with ALI cultures that supported parasite development. Further work to pinpoint specific conditions necessary to allow the completion of the parasite life cycle and expand them is required here. Stage-specific protein tagging either by CRISPR/Cas9-based methods (Tandel et al., 2019) or using newly-developed monoclonal antibodies (Wilke et al., 2018) can now assist in monitoring the life cycle progression of parasites in these *in vitro* models. Although both stem cell-based systems were able to demonstrate production of new oocysts, each suffered from diminishing returns that bar long-term cultivation. The inability to easily maintain transgenic parasite lines *in vitro* still poses a challenge to the *Cryptosporidium* field. With improvements that sustain higher oocyst yields, these new *in vitro* systems could possibly be adapted for the maintenance of transgenic parasite stocks, as well as other traditionally host-restricted *Cryptosporidium* species such as the anthroponotic *C. hominis*, which remains regrettably understudied.

*Cryptosporidium* research has been restricted by the lack of suitable model systems that accurately model infection and this has led to significant gaps in our understanding of infection and immunity. A major gap in our understanding is how immunity to *Cryptosporidium* infection is acquired and sustained. Further, the mechanisms behind growth stunting exhibited by afflicted infants are yet to be elucidated, as is the role of gut microbiota during and after infection. Parasite entry, replication, sexual reproduction, and oocyst release are yet to be truly visualized and studied at a molecular level. We believe these new *in vivo* and *in vitro* models can provide the basis for concerted explorations to expand our understanding of *Cryptosporidium* pathogenesis. By investing wisely in these new methods and pushing them to their maximal potentials, the *Cryptosporidium* and intestinal immunity research communities have much to gain.

## Author Contributions

NBM and AS wrote this manuscript together. All authors contributed to the article and approved the submitted version.

## Funding

This work was supported by funding from The Francis Crick Institute (https://www.crick.ac.uk/), which receives its core funding from Cancer Research UK, the UK Medical Research Council and the Wellcome Trust.

## Conflict of Interest

The authors declare that the research was conducted in the absence of any commercial or financial relationships that could be construed as a potential conflict of interest.

## References

[B1] Abrahamsen M. S. Templeton T. J. Enomoto S. Abrahante J. E. Zhu G. Lancto C. A. (2004). Complete Genome Sequence of the Apicomplexan, Cryptosporidium parvum. Science 304, 441–445. 10.1126/science.1094786 15044751

[B2] Amadi B. Mwiya M. Musuku J. Watuka A. Sianongo S. Ayoub A. (2002). Effect of nitazoxanide on morbidity and mortality in Zambian children with cryptosporidiosis: a randomised controlled trial. Lancet 360, 1375–1380. 10.1016/s0140-6736(02)11401-2 12423984

[B3] Aylor D. L. Valdar W. Foulds-Mathes W. Buus R. J. Verdugo R. A. Baric R. S. (2011). Genetic analysis of complex traits in the emerging Collaborative Cross. Genome Res. 21, 1213–1222. 10.1101/gr.111310.110 21406540 PMC3149489

[B4] Bar-Ephraim Y. E. Kretzschmar K. Clevers H. (2019). Organoids in immunological research. Nat. Rev. Immunol. 20, 279–293. 10.1038/s41577-019-0248-y 31853049

[B5] Choudhary H. H. Nava M. G. Gartlan B. E. Rose S. Vinayak S. (2020). A Conditional Protein Degradation System To Study Essential Gene Function in Cryptosporidium parvum. Mbio 11, e01231–e01220. 10.1128/mbio.01231-20 32843543 PMC7448269

[B6] Griffiths J. K. Theodos C. Paris M. Tzipori S. (1998). The Gamma Interferon Gene Knockout Mouse: a Highly Sensitive Model for Evaluation of Therapeutic Agents againstCryptosporidium parvum. J. Clin. Microbiol. 36, 2503–2508. 10.1128/jcm.36.9.2503-2508.1998 9705383 PMC105153

[B7] Heo I. Dutta D. Schaefer D. A. Iakobachvili N. Artegiani B. Sachs N. (2018). Modelling Cryptosporidium infection in human small intestinal and lung organoids. Nat. Microbiol. 3, 814–823. 10.1038/s41564-018-0177-8 29946163 PMC6027984

[B8] Jumani R. S. Hasan M. M. Stebbins E. E. Donnelly L. Miller P. Klopfer C. (2019). A suite of phenotypic assays to ensure pipeline diversity when prioritizing drug-like Cryptosporidium growth inhibitors. Nat. Commun. 10, 1862. 10.1038/s41467-019-09880-w 31015448 PMC6478823

[B9] Khalil I. A. Troeger C. Rao P. C. Blacker B. F. Brown A. Brewer T. G. (2018). Morbidity, mortality, and long-term consequences associated with diarrhoea from Cryptosporidium infection in children younger than 5 years: a meta-analyses study. Lancet Global Heal. 6, e758–e768. 10.1016/s2214-109x(18)30283-3 PMC6005120 29903377

[B10] Korpe P. S. Haque R. Gilchrist C. Valencia C. Niu F. Lu M. (2016). Natural History of Cryptosporidiosis in a Longitudinal Study of Slum-Dwelling Bangladeshi Children: Association with Severe Malnutrition. PloS Negl. Trop. D 10, e0004564. 10.1371/journal.pntd.0004564 PMC4856361 27144404

[B11] Kotloff K. L. Nataro J. P. Blackwelder W. C. Nasrin D. Farag T. H. Panchalingam S. (2013). Burden and aetiology of diarrhoeal disease in infants and young children in developing countries (the Global Enteric Multicenter Study, GEMS): a prospective, case-control study. Lancet 382, 209–222. 10.1016/s0140-6736(13)60844-2 23680352

[B12] Kváč M. McEvoy J. Loudová M. Stenger B. Sak B. Květoňová D. (2013). Coevolution of Cryptosporidium tyzzeri and the house mouse (Mus musculus). Int. J. Parasitol. 43, 805–817. 10.1016/j.ijpara.2013.04.007 23791796 PMC4437667

[B13] Love M. S. Beasley F. C. Jumani R. S. Wright T. M. Chatterjee A. K. Huston C. D. (2017). A high-throughput phenotypic screen identifies clofazimine as a potential treatment for cryptosporidiosis. PloS Negl. Trop. D 11, e0005373. 10.1371/journal.pntd.0005373 PMC5310922 28158186

[B14] Morales M. A. G. Ausiello C. M. Guarino A. Urbani F. Spagnuolo M. II Pignata C. (1996). Severe, Protracted Intestinal Cryptosporidiosis Associated with Interferon Deficiency: Pediatric Case Report. Clin. Infect. Dis. 22, 848–850. 10.1093/clinids/22.5.848 8722945

[B15] Pawlowic M. C. Somepalli M. Sateriale A. Herbert G. T. Gibson A. R. Cuny G. D. (2019). Genetic ablation of purine salvage in Cryptosporidium parvum reveals nucleotide uptake from the host cell. P Natl. Acad. Sci. U.S.A. 116, 21160–21165. 10.1073/pnas.1908239116 PMC6800313 31570573

[B16] Ren X. Zhao J. Zhang L. Ning C. Jian F. Wang R. (2012). Cryptosporidium tyzzeri n. sp. (Apicomplexa: Cryptosporidiidae) in domestic mice (Mus musculus). Exp. Parasitol. 130, 274–281. 10.1016/j.exppara.2011.07.012 21803038

[B17] Rossi G. Manfrin A. Lutolf M. P. (2018). Progress and potential in organoid research. Nat. Rev. Genet. 19, 671–687. 10.1038/s41576-018-0051-9 30228295

[B18] Sateriale A. Šlapeta J. Baptista R. Engiles J. B. Gullicksrud J. A. Herbert G. T. (2019). A Genetically Tractable, Natural Mouse Model of Cryptosporidiosis Offers Insights into Host Protective Immunity. Cell Host Microbe 26, 135–146.e5. 10.1016/j.chom.2019.05.006 31231045 PMC6617386

[B19] Sato T. Vries R. G. Snippert H. J. van de Wetering M. Barker N. Stange D. E. (2009). Single Lgr5 stem cells build crypt-villus structures in vitro without a mesenchymal niche. Nature 459, 262–265. 10.1038/nature07935 19329995

[B20] Slapeta J. (2011). Naming of Cryptosporidium pestis is in accordance with the ICZN Code and the name is available for this taxon previously recognized as C. parvum “bovine genotype”. Vet. Parasitol. 177, 1–5. 10.1016/j.vetpar.2011.01.049 21367525

[B21] Tandel J. English E. D. Sateriale A. Gullicksrud J. A. Beiting D. P. Sullivan M. C. (2019). Life cycle progression and sexual development of the apicomplexan parasite Cryptosporidium parvum. Nat. Microbiol. 4, 2226–2236. 10.1038/s41564-019-0539-x 31477896 PMC6877471

[B22] Upton S. J. Tilley M. Brillhart D. B. (1994). Comparative development of Cryptosporidium parvum (Apicomplexa) in 11 continuous host cell lines. FEMS Microbiol. Lett. 118 (3), 233–236. 10.1111/j.1574-6968.1994.tb06833.x 8020747

[B23] Vinayak S. Pawlowic M. C. Sateriale A. Brooks C. F. Studstill C. J. Bar-Peled Y. (2015). Genetic modification of the diarrhoeal pathogen Cryptosporidium parvum. Nature 523, 477–480. 10.1038/nature14651 26176919 PMC4640681

[B24] Wang X. Yamamoto Y. Wilson L. H. Zhang T. Howitt B. E. Farrow M. A. (2015). Cloning and variation of ground state intestinal stem cells. Nature 522, 173–178. 10.1038/nature14484 26040716 PMC4853906

[B25] Wang R.-J. Li J.-Q. Chen Y.-C. Zhang L.-X. Xiao L.-H. (2018). Widespread occurrence of Cryptosporidium infections in patients with HIV/AIDS: Epidemiology, clinical feature, diagnosis, and therapy. Acta Trop. 187, 257–263. 10.1016/j.actatropica.2018.08.018 30118699

[B26] Wilke G. Ravindran S. Funkhouser-Jones L. Barks J. Wang Q. VanDussen K. L. (2018). Monoclonal Antibodies to Intracellular Stages of Cryptosporidium parvum Define Life Cycle Progression In Vitro. Msphere 3, e00124–e00118. 10.1128/msphere.00124-18 29848759 PMC5976880

[B27] Wilke G. Funkhouser-Jones L. J. Wang Y. Ravindran S. Wang Q. Beatty W. L. (2019). A Stem-Cell-Derived Platform Enables Complete Cryptosporidium Development In Vitro and Genetic Tractability. Cell Host Microbe 26, 123–134.e8. 10.1016/j.chom.2019.05.007 31231046 PMC6617391

